# Clinical Indicators Distinguishing Pulmonary Tuberculosis from Community-Acquired Pneumonia in Older Adults: A Prospective Multicenter Study

**DOI:** 10.3390/pathogens15010033

**Published:** 2025-12-25

**Authors:** Mari Yamasue, Kosaku Komiya, Tetsuji Nakano, Ryosuke Hamanaka, Akihiko Goto, Shogo Ichihara, Takamasa Kan, Yuhei Nagaoka, Shoma Hirota, Yutaka Mukai, Ryohei Kudoh, Hiroaki Fujisawa, Ryota Moriyama, Atsushi Yokoyama, Takashi Yamamoto, Toshiko Ikebe, Seiya Kato

**Affiliations:** 1 Respiratory Medicine and Infectious Diseases, Faculty of Medicine, Oita University, 1-1 Idaigaoka, Hasama-machi, Yufu-shi 879-5593, Oita, Japan; 2 Internal Medicine, National Hospital Organization Nishi-Beppu Hospital, 4548 Tsurumi, Beppu-shi 874-0840, Oita, Japan; 3 Department of Respiratory Medicine, Tenshindo Hetsugi Hospital, 5956 Nakahetsugi-Kawadoko, Oita-shi 879-7761, Oita, Japan; 4 Department of Respiratory Medicine, Oita Medical Center, 2-11-45 Yokota, Oita-shi 870-0263, Oita, Japan; 5 Department of Respiratory Medicine, Oita Prefectural Hospital, 2-8-1 Hojyo, Oita-shi 870-8511, Oita, Japan; 6 Internal Medicine, Oita Memorial Hospital, 2-5-10 Haya Oita-shi 870-0854, Oita, Japan; 7 Respiratory Medicine, Oita Red Cross Hospital, 3-2-37 Chiyo-machi, Oita-shi 870-0033, Oita, Japan; 8 Internal Medicine, Usuki City Medical Association Hospital, 113-1 Tomuro-Nagatani, Usuki-shi 875-0051, Oita, Japan; 9 Department of Internal Medicine, Bungo-Ono City Hospital, 226 Baba, Ogata-machi, Bungo-Ono-shi 879-6692, Oita, Japan; 10 Public Health Policy and Infection Control Division, Oita Prefectural Government, 3-1-1 Ote-machi, Oita-shi 870-8501, Oita, Japan; 11 The Research Institute of Tuberculosis, Japan Anti-Tuberculosis Association, 3-1-24 Matsuyama, Kiyose-shi 204-8533, Tokyo, Japan

**Keywords:** pulmonary tuberculosis, community-acquired pneumonia, older adults, diagnosis

## Abstract

Clinical indicators for pulmonary tuberculosis (PTB) among patients with community-acquired pneumonia (CAP) have been derived from studies on younger or middle-aged populations in high TB-burden countries. However, diagnostic clues specific to older adults remain insufficiently validated. This multicenter prospective observational study aimed to identify the clinical features that can help differentiate PTB from CAP among older patients. We enrolled patients aged ≥ 65 years who were diagnosed with PTB or non-TB CAP between September 2023 and September 2025. Clinical data—including demographics, symptoms, and laboratory findings, previously reported as potential discriminators of PTB—were compared between the two groups. Of 233 patients included, 57 (24%) were diagnosed with PTB. No significant difference in sex was observed between the PTB and non-TB CAP groups. The PTB group was older and had a poorer performance status than the CAP group. On multivariate logistic regression analysis, PTB was significantly and independently associated with weight loss (aOR 8.17, *p* < 0.001); symptoms lasting ≥ 2 weeks (aOR 5.79, *p* < 0.001); and absence of general fatigue (aOR 0.19, *p* < 0.001) and dyspnea (aOR 0.19, *p* = 0.002) but not with night sweats and hemoptysis. These clinical features may be valuable indicators of PTB in older adults and inform tuberculosis control strategies in regions expected to have accelerated population aging.

## 1. Introduction

Tuberculosis (TB) remains one of the most significant infectious diseases worldwide from a public health perspective. With the global trend of population aging, the proportion of older individuals among patients with TB has been steadily increasing. According to international epidemiological studies, individuals aged ≥ 60 years account for approximately one-third of patients with newly diagnosed TB, representing an increase of approximately 26% since the 2010s [1]. In particular, in low-incidence countries in East Asia and Europe, the age distribution of patients with TB has shifted toward older populations [2,3,4]. Consequently, immunosenescence and the presence of multiple comorbidities have created new challenges in the diagnosis and treatment of TB among the elderly [5,6].

Japan was officially classified as a country with low TB incidence in 2021; however, more than 10,000 new cases remain reported annually. A distinctive feature of TB epidemiology in Japan is the remarkable preponderance in the very old; of all patients, approximately two-thirds are aged ≥ 65 years, 30% are in their 80s, and 14% are aged ≥ 90 years [7,8]. Moreover, mortality within 60 days of treatment initiation was reported to be markedly higher among older patients than in younger groups, and diagnostic delay has been identified as a major contributor to poor outcomes [9,10]. Therefore, early and accurate diagnosis is essential to improve prognosis and advance TB control in this age group.

Likewise, older individuals represent the predominant population affected by community-acquired pneumonia (CAP), which frequently needs to be differentiated from pulmonary TB (PTB) in clinical practice. Previous reports, including the Community-Acquired Pneumonia Organization cohort study that was conducted in the USA, have identified night sweats, weight loss, hemoptysis, history of TB exposure, and upper lobe predominance on chest imaging as predictive indicators of TB [11,12]. However, most of these studies were conducted in high-incidence settings or among younger and middle-aged adults, making the applicability of the same diagnostic markers to populations dominated by the very old, such as in Japan, uncertain. Indeed, older patients with PTB often lack classic symptoms, such as hemoptysis and night sweats, and present instead with nonspecific manifestations, such as fatigue and loss of appetite [13]. Similarly, radiographic findings may not display the typical upper lobe infiltrates or cavitary lesions [14], contributing to diagnostic delay. Reliable clinical findings that prompt suspicion of PTB in older people have not been well established. Therefore, this study aimed to identify the clinical symptoms and historical features that can be useful for differentiating PTB from non-TB CAP among older patients at the initial diagnosis stage.

## 2. Materials and Methods

### 2.1. Study Design and Participants

This multicenter prospective observational study enrolled inpatients and outpatients aged ≥ 65 years who were diagnosed with PTB or CAP other than TB (non-TB CAP) between September 2023 and September 2025 at Oita University Hospital and its nine collaborating institutions. The study protocol was approved by the ethics committee of Oita University Faculty of Medicine (approval number: 2631; approval date: 6 September 2023), and permission for implementation was obtained from the directors of all participating institutions. Written informed consent was obtained from each participant or their legal representative after providing a detailed explanation of the study objectives and procedures.

PTB was defined as the presence of respiratory symptoms, such as fever, productive cough, and sputum production, with radiographic evidence of pulmonary lesions and microbiological confirmation of *Mycobacterium tuberculosis* by culture (Mycobacteria Growth Indicator Tube; MGIT Becton, Dickinson and Company, Tokyo, Japan) or nucleic acid amplification testing of respiratory specimens (e.g., sputum, aspirated secretions, or gastric lavage). Non-TB CAP was defined as the presence of respiratory symptoms and radiographic pulmonary infiltrates; negative results of mycobacterial testing, including cultures; and clinical improvement after antimicrobial therapy other than anti-TB drugs. Patients without mycobacterial testing at diagnosis, those who developed hospital-acquired pneumonia ≥ 48 h after admission, and those diagnosed with non-TB mycobacterial disease were excluded.

### 2.2. Data Collection

To ensure standardized data acquisition across sites, a unified questionnaire (Supplemental Questionnaire) was used to record demographic and clinical information. Baseline data included disease classification (PTB or non-TB CAP), age, sex, height, weight, body mass index, performance status, level of consciousness (Japan Coma Scale ≥ II-10), presence of respiratory failure, prior history or exposure to TB, smoking and drinking habits, living environment (home vs. long-term care facility), and occupational history (healthcare or caregiving work). We also documented comorbidities, including chronic respiratory diseases, such as chronic obstructive pulmonary disease, interstitial pneumonia, and bronchiectasis; cardiac diseases, such as ischemic heart disease and heart failure; renal diseases, such as chronic kidney disease stage G5 or receipt of maintenance dialysis; hepatic dysfunction; cerebrovascular disease; dementia; diabetes mellitus (hemoglobin A1c ≥ 7%); collagen vascular disease; human immunodeficiency virus/acquired immunodeficiency syndrome; hematologic disorders; malignancy; history of gastrectomy or esophagectomy; and alcohol or substance dependence. Medication history included systemic corticosteroids, immunosuppressive agents, biologics, antineoplastic drugs, and acid suppressants. Clinical symptoms were collected according to previously reported predictive features of TB and included weight loss (duration and amount); emaciation (≥10% below ideal body weight); loss of appetite; general fatigue; night sweats; hemoptysis; hoarseness; dyspnea; and persistence of any symptom, such as fever, cough, and sputum, for ≥2 weeks.

Microbiological data included detected organisms and their quantity. Laboratory findings, including white blood cell count and differential, hemoglobin, C-reactive protein, aspartate aminotransferase, alanine aminotransferase, blood urea nitrogen, creatinine, albumin, N-terminal pro-B-type natriuretic peptide, interferon γ release assay, and anti-MAC antibody results, were collected. Radiographic information from chest X-ray and computed tomography and laboratory data were used to support PTB diagnosis but were not included in the statistical analysis. One participating facility declined to provide data on the laboratory results and chest imaging findings of its enrolled patients.

### 2.3. Statistical Analysis

Baseline characteristics and clinical symptoms were compared between the PTB and non-TB CAP groups. Sample size was calculated for binary outcomes using a two-sided ɑ of 0.05, power of 0.80, and an allocation ratio of 1:3 (PTB: non-TBCAP). Based on prior studies [11], which compared clinical characteristics between PTB and non-TB CAP, effect sizes of 0.30 and 0.10 were assumed for the PTB and non-TB CAP groups, respectively. Using Epitools (Sergeant, ESG, 2018. Epitools Epidemiological Calculators. Ausvet. Available at: http://epitools.ausvet.com.au. accessed on 8 May 2023). The sample size was estimated to be 176 patients in total (44 with PTB and 132 with non-TB CAP). Continuous variables were analyzed using a t-test after testing for normality, and categorical variables were analyzed using a chi-square test or Fisher’s exact test when the expected counts were <5. Variables that showed statistical significance on univariate analyses were entered into a multivariate logistic regression model to identify factors independently associated with TB diagnosis. However, we judged that the ascertainment of prior pulmonary tuberculosis and the presence of dementia based solely on patient interviews lacked sufficient reliability. Consequently, these variables were excluded from the multivariable analysis, for which the number of allowable covariates was constrained. Adjusted odds ratios (aORs) and 95% confidence intervals (CIs) were calculated with beta-values, and *p*-values < 0.05 were considered statistically significant. We also constructed a predictive model by scoring significant variables based on their β coefficients in the logistic regression analysis. Statistical analyses were performed using IBM SPSS Statistics version 25 (IBM Japan, Tokyo, Japan).

## 3. Results

Of 233 patients enrolled in this study, 57 (24%) were diagnosed with PTB, 43 (75%) of whom were smear-positive. Tests were positive for only culture in 7 (12%), only PCR in 23 (40%), and both culture and PCR in 27 (47%). In cases where only the tuberculosis PCR test yielded a positive result, the determination of active tuberculosis was made in conjunction with the clinical presentation. No significant differences were observed between the PTB and non-TB CAP groups in terms of sex. Compared with the non-TB CAP group, the PTB group was older and had poorer performance status. In addition, the PTB group was more likely to have a previous history of TB (15.8% vs. 1.1%, *p* < 0.001); comprised less patients with dementia (9.1% vs. 29.7%, *p* = 0.002); and had significantly more frequent clinical manifestations, such as weight loss (56.1% vs. 14.8%, *p* < 0.001), emaciation (43.9% vs. 25.6%, *p* = 0.009), and symptom duration ≥ 2 weeks (56.1% vs. 23.9%, *p* < 0.001). The median duration of weight loss was 6 months, with a median loss of 5 kg (interquartile range, 3–5 kg). Conversely, symptoms that were traditionally considered characteristic of PTB, such as night sweats and hemoptysis, did not differ significantly between groups. Compared with the PTB group, the non-TB CAP group more frequently reported general fatigue (65.3% vs. 29.8%, *p* < 0.001) and dyspnea (35.8% vs. 12.3%, *p* = 0.001) (Table 1). Available laboratory data and radiological findings among 209 patients are shown in Supplementary Table S1.

**Figure 1:**
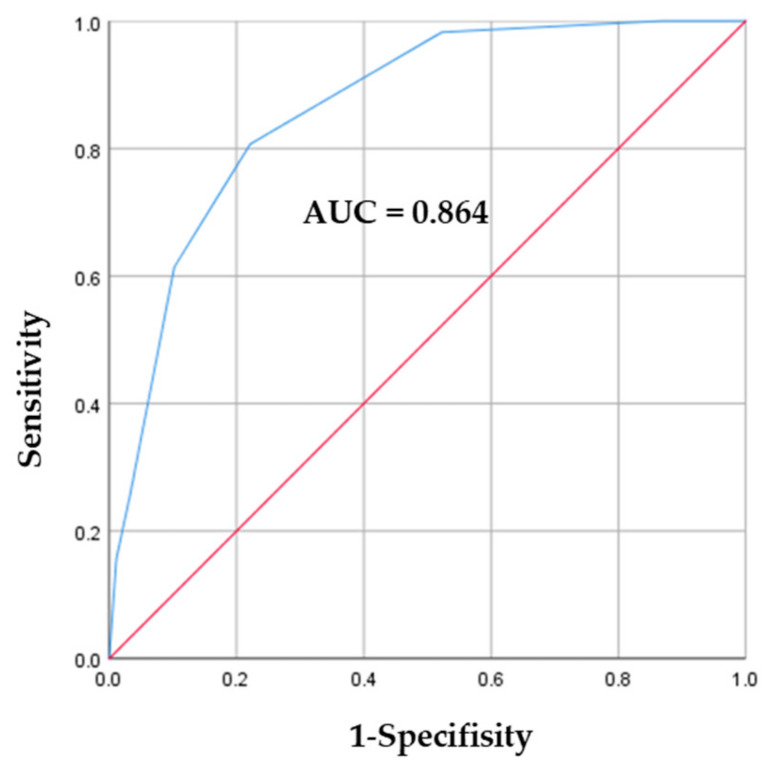
Receiver operating characteristic (ROC) curve (blue curve) and area under the curve (AUC) of the prediction model derived from four symptom items: weight loss, absence of general fatigue, absence of dyspnea, and symptoms lasting ≥ 2 weeks.

On multivariate logistic regression analysis, the following four factors were independently associated with PTB diagnosis: weight loss (adjusted OR 8.17, 95% CI 3.53–18.91), symptom duration ≥ 2 weeks (adjusted OR 5.79, 95% CI 2.52–13.33), absence of general fatigue (adjusted OR 0.19, 95% CI 0.08–0.43), and absence of dyspnea (adjusted OR 0.19, 95% CI 0.06–0.55) (Table 2). Based on the β coefficients, we assigned a score of 2 points for weight loss, 1 point for absence of general fatigue, 1 point for absence of dyspnea, and 2 points for symptoms persisting for ≥2 weeks (Table 3). The AUC of the scoring model was 0.864 (95% CI: 0.814–0.914) (Figure 1). When the cutoff value of the symptom score was set at 3 points, the sensitivity and specificity were 0.807 and 0.790, respectively, with a positive predictive value of 0.554 and a negative predictive value of 0.927.

Although the present study focused on the clinical course and symptoms suggestive of pulmonary tuberculosis, chest imaging and blood test results were available for 34 of 57 patients (59.6%) in the pulmonary tuberculosis group and for 175 of 176 patients (99.4%) in the non-tuberculous community-acquired pneumonia group. When comparing only these subsets, the non-tuberculous community-acquired pneumonia group demonstrated higher white blood cell counts, CRP, and albumin levels, whereas nodules, cavities, and pleural effusion were more frequently observed in the pulmonary tuberculosis group (Supplementary Table S1).

## 4. Discussion

In this multicenter prospective study on elderly patients, weight loss, persistence of symptoms for ≥2 weeks, and absence of general fatigue and dyspnea were identified as clinical features independently associated with PTB. Conversely, symptoms of night sweats and hemoptysis, which were traditionally regarded as characteristic of PTB, were not useful discriminators among the older population.

The diagnostic value of weight loss and prolonged symptoms in older adults may reflect the interplay between immunosenescence and chronic low-grade inflammation or inflammaging. Immunosenescence involves a decline in naïve T cells, clonal expansion of memory T cells, and dysregulated cytokine production [15,16], leading to attenuated Th1 responses and impaired interferon γ secretion in response to *M. tuberculosis* [17]. This delayed macrophage activation contributes to persistent infection and chronic inflammation. Sustained elevations in proinflammatory cytokines, such as TNFɑ, IL6, and IL1β, promote catabolism and muscle breakdown [18,19], which may become clinically evident as weight loss among older individuals, who often have baseline frailty, malnutrition, or sarcopenia [20]. In addition, the indolent course of infection aligns with the prolonged symptom duration in our cohort. The relatively low prevalence of general fatigue in the PTB group likely reflects a relative difference in inflammatory dynamics between PTB and non-TB CAP. The rapid increase in IL1β, IL6, and TNFɑ induced by acute bacterial pneumonia leads to hypermetabolism and marked fatigue through accelerated protein catabolism [21]. However, within such a short time frame, these symptoms may not have sufficient impact to result in clinically meaningful weight loss. In contrast, TB triggers a slower and localized inflammatory response, with gradual cytokine elevation that may not provoke acute systemic malaise [22]. Age-related alterations in cytokine responsiveness and diminished acute-phase reactivity [23] may further attenuate symptom perception in elderly patients with TB. Similarly, the lower frequency of dyspnea among elderly patients with PTB than in those with non-TB CAP may reflect differences in the distribution and extent of parenchymal involvement. PTB typically produces localized lesions, such as cavitation, centrilobular nodules, and tree-in-bud patterns, and usually involves fewer lobes, whereas non-TB CAP often causes alveolar consolidation in the middle to lower lung fields, which can readily progress to multilobar or bilateral involvement [14]. These differences in lesion characteristics are thought to be related with the severity and rapidity of gas exchange impairment and may partly explain the less apparent symptoms in patients with TB.

The lack of diagnostic utility for night sweats and hemoptysis in the elderly may be explained by age-related physiological changes and comorbidities. Night sweats result from autonomic thermoregulatory responses, which are blunted in older adults due to decreased sympathetic activity and impaired autonomic control [24]. Regarding hemoptysis, weakened cough reflexes and impaired expectoration [25] make sputum less noticeable; in elderly TB, apical cavitary lesions are less common and peripheral infiltrates predominate [14]. These factors collectively reduce the likelihood of classical TB symptoms manifesting in this age group.

A major strength of this study is its prospective, real-world comparison between PTB and CAP, specifically among elderly patients. The findings highlighted that even in the absence of typical radiological features, which are often atypical in older adults [14], simple clinical cues, such as weight loss and persistent symptoms, can play a critical role in prompting TB suspicion and facilitating early microbiological testing. This pragmatic insight aligns with daily clinical practice, when symptom recognition guides the decision to perform diagnostic testing. However, certain limitations should be acknowledged. First, owing to the observational nature of the study, potential selection and information biases cannot be fully excluded. Symptom assessment inevitably involved subjective interpretation, and interobserver variability may have influenced data accuracy. Second, this analysis focused primarily on medical history and clinical symptoms and did not incorporate laboratory or imaging findings into the predictive model. Nevertheless, given that clinical suspicion based on symptoms typically precedes diagnostic testing in real-world settings, this approach remains clinically relevant. Finally, this multicenter study was conducted within a single prefecture; thus, regional variations in healthcare accessibility may limit the generalizability of our results to the entire Japanese population.

In conclusion, weight loss, a symptom duration of ≥2 weeks, and the absence of general fatigue and dyspnea may serve as potential clinical indicators in distinguishing PTB from CAP among elderly patients. Conversely, traditional hallmark symptoms, such as night sweats and hemoptysis, may be less informative in this population. Considering the ongoing trend of population aging, these findings may provide preliminary insights into early TB detection strategies not only in Japan but also in countries undergoing similar demographic transitions. Further validation in external cohorts will be important to determine whether a diagnostic model tailored to the elderly population can be established.

## Figures and Tables

**Table 1:** Univariate analysis of the baseline characteristics of patients with pulmonary tuberculosis at diagnosis compared with those with community-acquired pneumonia.

	PTB (n = 57)	CAP (n = 176)	*p*
Female sex	30 (52.6)	80 (45.5)	0.346
Age, years	86 (78, 90)	82 (76, 87)	0.045
PS	3 (1, 3)	2 (1, 3)	0.020
BMI, kg/m^2^	19.3 (17.0, 22.2)	20.1 (17.1, 23.1)	0.142
Healthcare worker	7 (12.3)	15 (8.5)	0.399
Resident of medical and nursing care facility	11 (19.3)	39 (22.2)	0.647
Smoker	24 (42.1)	85 (48.3)	0.416
Asthma	6 (10.5)	31 (17.6)	0.203
COPD	10 (17.5)	44 (25.0)	0.246
Interstitial lung disease	8 (14.0)	15 (8.5)	0.225
Bronchiectasis	2 (3.5)	14 (8.0)	0.369
Old pulmonary tuberculosis	9 (15.8)	2 (1.1)	<0.001
Malignancy	7 (12.3)	21 (11.9)	0.994
Heart failure	15 (26.3)	68 (38.6)	0.091
CKD	15 (26.3)	29 (16.5)	0.099
Hepatic diseases	2 (3.5)	6 (3.4)	1.000
Cerebrovascular disease	11 (19.3)	39 (22.2)	0.647
Dementia	5 (9.1)	52 (29.7)	0.002
Substance use disorder	0 (0.0)	1 (0.6)	1.000
Diabetes mellitus	15 (26.3)	33 (18.8)	0.220
HIV	0 (0)	0 (0)	—
Hematologic disorders	1 (1.8)	2 (1.1)	0.571
Autoimmune disease	2 (3.5)	16 (9.1)	0.254
Immunosuppressant use	10 (17.5)	22 (12.5)	0.336
Anticancer drug use	1 (1.8)	4 (2.3)	1.000
History of esophagogastrectomy	5 (8.8)	6 (3.4)	0.143
Antacid use	21 (36.8)	77 (43.8)	0.359
Disturbance of consciousness	7 (12.3)	13 (7.4)	0.278
Weight loss	32 (56.1)	26 (14.8)	<0.001
Emaciation (−10% of IBW)	25 (43.9)	45 (25.6)	0.009
Lack of appetite	21 (36.8)	88 (50.0)	0.084
General fatigue	17 (29.8)	115 (65.3)	<0.001
Night sweats	4 (7.0)	13 (7.4)	1.000
Hemoptysis	2 (3.5)	15 (8.5)	0.255
Hoarseness	3 (5.3)	17 (9.7)	0.418
Dyspnea	7 (12.3)	63 (35.8)	0.001
Symptoms lasting ≥2 weeks	32 (56.1)	42 (23.9)	<0.001

Data are presented as numbers (%) or median (interquartile range). PS: performance status, BMI: body mass index, COPD: chronic obstructive pulmonary disease, CKD: chronic kidney disease, HIV: human immunodeficiency virus, IBW: ideal body weight.

**Table 2:** Multivariate analysis of the baseline characteristics of patients with pulmonary tuberculosis at diagnosis compared with those with community-acquired pneumonia.

	β	OR	95% CI	*p*
Age, years	0.005	1.005	0.952–1.061	0.851
PS	0.104	1.110	0.766–1.608	0.582
Weight loss	2.100	8.166	3.526–18.912	<0.001
Emaciation (−10% of IBW)	0.134	1.144	0.469–2.791	0.768
General fatigue	−1.678	0.187	0.082–0.426	<0.001
Dyspnea	−1.682	0.186	0.063–0.546	0.002
Symptoms lasting ≥2 weeks	1.756	5.792	2.517–13.331	<0.001

IBW: ideal body weight, PS: performance status.

**Table 3:** Symptom scoring model for diagnosis of pulmonary tuberculosis in older adults.

	Score
Weight loss	2
Absence of General fatigue	1
Absence of Dyspnea	1
Symptoms lasting ≥2 weeks	2

## Data Availability

Data are available from the corresponding author upon reasonable request.

## Supplementary Materials

The following supporting information can be downloaded at https://www.mdpi.com/article/10.3390/pathogens15010033/s1, Supplemental Questionnaire—version 3.0., created on 1 September 2023; Table S1: Comparison of laboratory and chest X-ray findings between elderly patients with pulmonary tuberculosis and those with non-tuberculous community-acquired pneumonia at diagnosis.

## Abbreviations

The following abbreviations are used in this manuscript:

CAP	Community-acquired pneumonia
CI	Confidence interval
CKD	Chronic kidney disease
OR	Odds ratio
