# Supplementary material for: Clinical Indicators Distinguishing Pulmonary Tuberculosis from Community-Acquired Pneumonia in Older Adults: A Prospective Multicenter Study
Source: Pathogens. 2025 Dec 25;15(1):33. doi: 10.3390/pathogens15010033 (PMC12845188; doi:10.3390/pathogens15010033)
Supplement: Supplementary file 1 [file pathogens-15-00033-s001.zip › pathogens-4056297-supplementary.pdf]

Supplementary materials

Questionnaire (Version 3.0), Created on September 1, 2023
Study on Early Diagnosis of Pulmonary Tuberculosis in Elderly Patients
— Usefulness of Predictive Factors for Pulmonary Tuberculosis in the Elderly —

Please answer the following questions.
For multiple-choice questions, please circle the appropriate answer.
For open-ended questions, please write your response in the boxes provided.

1. Sex
   Male ・ Female
2. Age
   (   ) years
3. Height / Weight
   Height (   ) cm ・ Weight (   ) kg
4. Diagnosis
If unsure, please ask your physician or public health nurse.
   Community-acquired pneumonia ・ Pulmonary tuberculosis
   → If pulmonary tuberculosis: Smear-positive ・ Smear-negative
5. Have you ever had tuberculosis in the past?
   Yes ・ No
   → If yes: With treatment ・ Without treatment
6. Have you ever been in contact with a person with tuberculosis?
   Yes ・ No
7. Comorbidities
  Please select all that apply.
  Chronic respiratory diseases:
  Asthma, Chronic obstructive pulmonary disease (COPD), Interstitial lung disease (including silicosis),
  Bronchiectasis
  Cardiovascular diseases: Angina pectoris / Myocardial infarction, Heart failure
  Kidney disease
   → If yes:
   Stage 5 chronic kidney disease: Yes ・ No
   Maintenance dialysis: Yes ・ No
Liver disease
  Cerebrovascular disease (cerebral infarction / cerebral hemorrhage), Dementia
  Diabetes mellitus
   → Most recent HbA1c (   )%
  Collagen vascular disease or other autoimmune diseases
  HIV infection / AIDS
  Hematologic diseases
  Malignancy
  History of gastrectomy or esophagectomy
   Select if you have had surgery on the stomach or esophagus.

8. Respiratory failure at diagnosis
Present ・ Absent
Definition: $SpO_2 < 90\%$ on room air or requiring supplemental oxygen to maintain $SpO_2 \geq 90\%$

9. Smoking history
Yes ・ No
→ If yes: from age (   ) to age (   ), (   ) cigarettes/day

10. Alcohol consumption
Yes ・ No
→ If yes: Regularly ・ Occasionally (less than once per week)

11. Medication use
Please select all that apply.
Steroids
Immunosuppressants
Biologics (Medication name:)
Please write the name if known.
Anticancer drugs (for patients undergoing chemotherapy)
→ Cytotoxic agents ・ Molecular-targeted agents ・ Immune checkpoint inhibitors
Select if known.
Antacid medications (acid-suppressing drugs)

12. Alcohol or drug dependence
Present ・ Absent

13. Primary living setting
Home ・ Medical / long-term care facility

14. Are you currently working?
Yes ・ No
→ If yes:
Is your work related to healthcare or caregiving? Yes ・ No

15. Symptoms
Please select all that apply.
Weight loss
→ If yes: Lost (   ) kg over (   ) months
More than 10% loss from ideal body weight
Loss of appetite
Fatigue / general malaise
Night sweats
Hemoptysis
Hoarseness
Dyspnea (shortness of breath)
Symptoms lasting for more than 2 weeks (cough, fever)
→ If yes: Cough ・ Fever ・ Both

End of Questionnaire

Thank you very much for your cooperation.

Supplementary Table S1. Comparison of laboratory and chest X-ray findings between elderly patients with pulmonary tuberculosis and those with non-tuberculous community-acquired pneumonia at diagnosis.

	PTB (n = 34)	CAP (n = 175)	*P*
WBC ($\times 10^3/\mu L$)	6.6 (5.2–8.2)	10.4 (7.1–13.8)	0.001
Hemoglobin (g/dL)	11.8 (10.8–13.0)	11.6 (10.3–12.3)	0.253
CRP (mg/dL)	3.2 (1.1–7.6)	9.0 (5.2–16.0)	<0.001
Albumin (g/dL)	2.8 (2.1–3.5)	3.1 (2.7–3.5)	0.012
AST (IU/L)	27 (20–42)	25 (18–39)	0.994
ALT (IU/L)	18 (10–23)	17 (11–29)	0.836
BUN (mg/dL)	17.3 (13.8–21.6)	18.6 (13.9–25.9)	0.154
Creatinine (mg/dL)	0.73 (0.57–0.87)	0.78 (0.64–1.16)	0.079
Consolidation	26 (76.5)	151 (86.3)	0.146
Nodule	20 (58.8)	9 (5.1)	< 0.001
Cavity	13 (38.2)	6 (3.4)	< 0.001
Pleural effusion	13 (38.2)	38 (21.7)	0.040
Distribution			
Bilateral lungs	18 (52.9)	95 (54.3)	0.886
Lateral lung	16 (47.1)	79 (45.1)	0.837
Right lung	19 (55.9)	88 (50.3)	0.550
Upper fields	10 (29.4)	28 (16.0)	0.064

Data are presented as the number (%) or median (interquartile range).

ALT, alanine aminotransferase; AST, aspartate aminotransferase; BUN, blood urea nitrogen; CAP, community-acquired pneumonia; CRP, C-reactive protein; PTB, pulmonary tuberculosis; WBC, white blood cell
